# Complete Genome Sequence of a Very Virulent Porcine Epidemic Diarrhea Virus Strain, CH/GDGZ/2012, Isolated in Southern China

**DOI:** 10.1128/genomeA.00645-13

**Published:** 2013-08-22

**Authors:** Ye Tian, Danping Su, Haiming Zhang, Rui-ai Chen, Dongsheng He

**Affiliations:** College of Veterinary Medicine, South China Agricultural University, Tianhe District, Guangzhou, Guangdong, People’s Republic of Chinaa; Guangdong Enterprise Key Laboratory of Biotechnology R&D of Veterinary Biologics, Guangdong, Zhaoqing, Chinab

## Abstract

The classical symptoms of porcine epidemic diarrhea (PED) are acute diarrhea and dehydration. The isolated porcine epidemic diarrhea virus (PEDV) CH/GDGZ/2012 strain was obtained from the feces of diseased pigs in 2012 in southern China. We report the complete genome sequence of strain CH/GDGZ/2012, which might be useful for better understanding the molecular characteristics of this virus.

## GENOME ANNOUNCEMENT

Porcine epidemic diarrhea virus (PEDV), which is an enveloped, positive-sense, single-stranded RNA virus, is classified as a member of the genus *Alphacoronavirus*, the family *Coronaviridae*, and the order *Nidovirales* (1, 2).

The whole genome of the PEDV strain CH/GDGZ/2012 was amplified by 21 sets of primers (3) and cloned into the pMD18-T vector (TaKaRa Bio, Inc., Japan), followed by sequencing twice by Beijing AuGCT DNA-SYN Biotechnology Co., China. The DNA sequences were assembled using DNAStar (version 7.1). Multiple-sequence alignment was performed with ClustalX (BioEdit version7). A phylogenetic tree was constructed for genome sequences using the MEGA 5.1 program.

The complete genome sequence of CH/GDGZ/2012 exhibits 96.6%, 97.4%, 97.9%, and 97.0% nucleotide homologies with the genomes of PEDV strains CV777, DR13, CH/FJND-3/2011, and CH/S, respectively (3–6). Among the genes, the S gene of CH/GDGZ/2012 has 93.6% to 97.1% nucleotide sequence identity with those of the strains reported previously. It is significant that there were two insertions (nucleotides [nt] 20824 to 20834 and 21068 to 21070) and three deletions (nt 21120 to 21122, 21130 to 21131, and 24243 to 24245) compared to strains DR13 and CV777 (3, 5). Similar mutations may also be found in the S gene of PEDV strains of GD-A and CH/S, which were recently isolated in China. The open reading frame 3 (ORF3) of CH/GDGZ/2012, which encodes the only accessory protein related to virulence (7), has 96.1% to 97% nucleotide sequence identity with the corresponding ORFs of the reported strains. Phylogenetic analysis of the complete genome showed that several PEDVs isolated in southern China belong to the same subgroup, but the classical vaccine strains CV777 and DR13 belong to another subgroup. There are 5 amino acid substitutions (L502S, S516A, T548S, G593S, and Q632E) in the strain.

These data may be useful for analyses of the epidemiology and evolutionary characteristics of PEDVs in southern China.

### Nucleotide sequence accession number. 

The complete genome sequence of PEDV CH/GDGZ/2012 strain has been deposited in GenBank under the accession no. KF384500.
